# Necessity of Internal Monitoring for Nuclear Medicine Staff in a Large Specialized Chinese Hospital

**DOI:** 10.3390/ijerph13040418

**Published:** 2016-04-12

**Authors:** Hong-Bo Wang, Qing-Zhao Zhang, Zhen Zhang, Chang-Song Hou, Wen-Liang Li, Hui Yang, Quan-Fu Sun

**Affiliations:** 1 Key Laboratory of Radiological Protection and Nuclear Emergency, National Institute for Radiological Protection, Chinese Center for Disease Control and Prevention, Beijing 100088, China; hongpo212@163.com (H.-B.W.); zhangqingzhao83@163.com (Q.-Z.Z.); zhangzhen04@126.com (Z.Z.); cdc_changsong@163.com (C.-S.H.); 2 Graduate School of Chinese Center for Disease Control and Prevention, Beijing 100088, China; 3 Henan Tumor Hospital, Zhengzhou 450008, China; henanzl@126.com (W.-L.L.); 13938276142@163.com (H.Y.)

**Keywords:** nuclear medicine, internal monitoring, necessity

## Abstract

This work intends to quantify the risk of internal contaminations in the nuclear medicine staff of one hospital in Henan province, China. For this purpose, the criteria proposed by the International Atomic Energy Agency (IAEA) to determine whether it is necessary to conduct internal individual monitoring was applied to all of the 18 nuclear medicine staff members who handled radionuclides. The activity of different radionuclides used during a whole calendar year and the protection measures adopted were collected for each staff member, and the decision as to whether nuclear medicine staff in the hospital should be subjected to internal monitoring was made on the basis of the criteria proposed by IAEA. It is concluded that for all 18 members of the nuclear medicine staff in the hospital, internal monitoring is required. Internal exposure received by nuclear medicine staff should not be ignored, and it is necessary to implement internal monitoring for nuclear medicine staff routinely.

## 1. Introduction

According to census data of the Chinese Society of Nuclear Medicine in 2009, 2011 and 2013 [1,2,3], radionuclide therapy has been developing very rapidly, and staff engaged in the work of nuclear medicine has also increased in recent years. By the end of 2013 [3], there were 610 medical institutions engaged in radionuclide therapy and a total of 8678 workers were working in the field of diagnostic and therapeutic nuclear medicine in China.

Practices in nuclear medicine involve the manipulation of many radiopharmaceuticals labeled with different unsealed radionuclides, both for diagnostic and therapeutic purposes, and may pose significant risks of internal contaminations to the workers. From a radiation protection point of view, the accurate assessment of the dose of internal exposure that nuclear medicine staff receives is as important as the accurate assessment of the dose of external exposure. Although it is considered that doses from radionuclide intakes in nuclear medicine centers are usually lower than doses from external exposure, the risks related with intakes should be estimated in every case and, if it is necessary, workers involved in the handling of unsealed sources should be monitored routinely, ensuring that the doses that workers receive are maintained as low as reasonably achievable. Internal monitoring should also be performed in case of accident or when suspected inhalation or ingestion intake occurs.

According to the 2014 Basic Safety Standards [4], routine internal monitoring should be conducted on workers in controlled areas where there are risks associated with incorporation of radionuclides. ICRP in its publication 78 [5] recommends that thyroid monitoring should be carried out in interval of 7, 14 or 30 days for those workers engaged in iodine therapy, depending on the workload, sensitivity of the detection system and the methodology available for the implementation of a monitoring program. Relevant standards in China [6,7] only stipulate a general and vague principle that workers engaged in producing a large number of radioactive isotopes and processing a large number of ^131^I labeled radiopharmaceuticals, and workers possibly internally contaminated by radioactive material, should be subjected to conventional internal individual monitoring. The internal monitoring of nuclear medicine staff, however, has not been carried out in most institutions in China thus far. The need for individual internal monitoring is dependent on the quantity and type of radioactive material present, the physical and chemical form of the radioactive material, the type of containment used, the practices implemented, as well as the general working conditions.

It is rather difficult to determine whether it is necessary to conduct internal monitoring for intakes of radioactive material for a worker. In 1999, the International Atomic Energy Agency (IAEA) published a safety guide (No.RS-G-1.2) [8] which aims to provide a set of criteria to be taken into account in order to determine whether or not an internal monitoring program is needed. The criteria are based on the evaluation of several factors to estimate the dose due to intake of radionuclides in the workplace. The decision on implementing internal monitoring is carried out when the evaluation results in an annual committed effective dose equal to or higher than 1 mSv.

In recent years, more and more attention has been focused on the internal exposure of nuclear medicine staff. Research in this area is divided into two parts: the first part is the study of the committed effective dose resulting from intake of radionuclides. A series of studies have been carried out on the internal exposure of nuclear medical staff by air sampling [9,10,11,12,13], direct measurement [14,15,16,17,18] or biological materials sample analysis [17,19,20]. The second part is the study of the necessity of internal monitoring for nuclear medicine staff. Researchers in Chile [21], Portugal [22], Brazil [23] have carried out some research based on the aforementioned methodology to determine whether staff should be subjected to internal monitoring. Since the 1980s, there have been only a few studies and a small number of reports on the work of internal exposure in the field of nuclear medicine in China [9,18]. This work reports the application of criteria proposed by the IAEA to determine for the first time whether internal monitoring is needed for nuclear medicine staff in a Chinese hospital.

## 2. Methods

### 2.1. Criterion for Judgement of Internal Monitoring

The criteria proposed by the IAEA in 1999 to assess whether internal individual monitoring is necessary is based on the potential for committed effective doses of 1 mSv or greater in one year, and based on the calculation of the “decision factor” (d*_j_*), which takes into account safety factors related with the complexity of the practice, handling conditions of the material, as well as the physical and chemical properties of the material being handled.

IAEA criteria can be briefly described as follows: internal monitoring is necessary whenever d*_j_* is equal to or higher than 1 mSv. The value of d*_j_* is calculated by the following expression (Equation (1)):

(1) $$d_{j}=\frac{A_{j}e{\left(g\right)}_{j,inh}f_{fs}f_{hs}f_{ps}}{0.001}$$

where *A_j_* is the average annual activity (Bq) of radionuclide *j* handled by a worker; e(g)*_j,inh_* is the dose coefficient for inhalation of 5 μm aerosols of radionuclide *j* by a worker (Sv·Bq^−1^);The physical form safety factor *f_fs_* is a factor associated with the physical and chemical properties of the material being handled. In most cases, *f_fs_* should be given a default value of 0.01. However, sometimes, a value of 0.001 can be used when it can be justified. The handling safety factor *f_hs_* is a factor associated with experience of the operation being performed and the form of the material. The protection safety factor *f_ps_* is a factor associated with the types of protective equipment employed (e.g., fume hood); 0.001 is a conversion factor from Sv to mSv.

If *f_fs_* adopts the default value of 0.01, the above Equation (Equation (1)) may be simplified to(Equation (2)):

(2) $$d_{j}=10A_{j}e{\left(g\right)}_{j.inh}f_{hs}f_{ps}$$

The decision factor for all radionuclides handled by workers in the workplace is the sum of all d*_j_* factors. Therefore, the total decision factor D is calculated according to the following expression (Equation (3)):

(3) $$D=\sum_{j}d_{j}$$

Then, if D is 1 or more, a need for individual monitoring would be demonstrated, and if D is less than 1, individual monitoring may be unnecessary.

If more than one radionuclide is handled in the workplace, decisions of conducting individual monitoring for each radionuclide may be based on the following criteria:

(1) all radionuclides for which d*_j_* ≥ 1 shall be monitored;
(2) when D ≥ 1, radionuclides for which d*_j_* ≥ 0.3 should be monitored;
(3) monitoring of radionuclides for which d*_j_* is much less than 0.1 is not necessary.

The dose coefficients used in the decision factor calculations originated from the biokinetic models of the International Commission on Radiological Protection Publication 68 [24]. Table 1 presents the dose coefficients for inhalation of 5 μm aerosols by workers for the commonly handled radionuclides. Handling safety factor (*f_hs_*) and protection safety factor (*f_ps_*) listed in Table 2 and Table 3 are defined based on the IAEA criteria [8], and on the values reported by Navarro [25].

### 2.2. Collection of Data

The rotation of work, the type, frequency and the protection measures for the operation of the radionuclide of each staff were obtained through field investigation and face-to-face interview of the workers using 3-page questionnaire.

The annual handled ^131^I activity was estimated based on the typical activity per patient and the recorded annual total numbers of patients treated with ^131^I. Additionally, the daily reports of the last two months were obtained to determine the handled activity of the ^99m^Tc per week and per year.

## 3. Results

### 3.1. Collected Information

The Henan Tumor Hospital is located in Zhengzhou, the provincial capital of Henan Province in east-central China. Transport in Zhengzhou is convenient, and the hospital is large and specialized in cancer diagnosis and tertiary treatment, so people from various regions visit the hospital. One thousand and four hundred cases of hospitalized patients with thyroid cancer were treated and nearly 20,000 person-time of imaging examination were performed in the nuclear medicine department in 2014, which are in lead in China. Currently, 18 workers in the department handle large amounts of radiopharmaceuticals, which presents workers at higher risk of intake of radionuclides.

There were 18 staff members in the nuclear medicine department in this hospital, including three technicians, nine doctors and six nurses. Nine doctors and three technicians took turns performing the procedure of ^99m^Tc elution, labelling and dose fractionation, one person per week. Six nurses took turns performing the procedure of ^99m^Tc injection, one person per week. From the daily reports of the last two months, we calculated the weekly handled activity and concluded that there is no significant change between weeks. In order to simplify the calculation, we divided the annual activity of ^99m^Tc elution, labelling and dose fractionation by 12 and obtained the annually corresponding handled activity of each worker. In the same way, by dividing the annual activity of ^99m^Tc injection by 6, we got the annual injection activity of each worker.

The selected nuclear medicine department treated 40 patients with thyroid cancer at the same time, as a batch, and over the past year, the department treated a total of 35 batches of patients with thyroid cancer. The 40 patients of each batch were hospitalized and treated in isolation and released after a week of treatment at the same day. Nine doctors and three technicians took turns performing the procedure of ^131^I automatic dose fractionation for the treatment of thyroid cancer, one person per batch. We divided the annual activity of automatic ^131^I dose fractionation by 12 and obtained the annually fractionated activity of each worker.

Table 4 shows the collected information in the selected specialized hospital.

The handling safety factors (*f_hs_*) and protection safety factors (*f_ps_*) values assigned to each identified operation varied depending on the circumstances of the operations performed. They are listed in Table 5. After obtaining relevant factors, we applied the proposed IAEA criteria and calculated the decision factor according to Equations (2) and (3).

### 3.2. Judgment of Necessity

Depending on the annual handled activities and on all the aforementioned factors, decision factor values (d*_j_*) for each operation have been obtained. The total decision factor D for each worker has also been calculated.

Table 6 presents all the procedures performed by each worker, annual activity of each procedure, d*_j_* value of each procedure and D value of each worker in the studied nuclear medicine department. It is quite obvious that d*_j_* of ^99m^Tc and ^131^I of each worker, and factor D of each worker are all higher than 1, and hence a routine internal monitoring are needed for all the 18 staff, especially for nine doctors and three technicians handling ^131^I as a result of the large workload.

## 4. Discussion

All staff members of nuclear medicine in a specialized provincial cancer hospital were interviewed, radionuclides handling data were obtained to determine the necessity of internal monitoring. Due to the large workload in this hospital, it was found to be necessary to monitor all 18 staff members.

There are many similar studies in other countries. Dantas *et al*. [23] observed that ^131^I presents a high risk, and internal monitoring of ^131^I should be included in the radiological protection plan, especially when used in therapeutic applications where the quantity of activity administered to each patient is usually several tens of millicuries. Bento *et al*. [22] found that 71.9% of all staff from nuclear medicine centers from all the participating institutions should be integrated in a routine monitoring program for internal contaminations. Astudillo *et al*. [21] also conducted a similar research in three nuclear medicine centers on the basis of IAEA criteria, and found it is necessary to carry out a routine monitoring program for five workers who handle ^131^I and three workers who handle ^99m^Tc, where there were in total seven workers who handled ^131^I and five workers who handled ^99m^Tc. Hence more than half of the workers investigated should be subjected to internal monitoring.

This study shows that it is necessary to carry out routine internal monitoring for all of the 18 staff members. The ratio might be higher compared with other studies, highlighting the necessity of internal monitoring and importance of internal contamination. To the best of our knowledge, this work is the first of its kind to determine whether internal monitoring is needed for nuclear medicine staff in a Chinese hospital by applying the criteria proposed by the IAEA in 1999, and so fills a gap in China.

It should be noted, however, that the decision factor values related to a certain operation, and units of mSv do not represent the dose truly received by the worker performing such an operation but represent a potential committed effective dose that can occur whilst performing that operation. As can be seen from Equation (2), the greater activity handled by the worker (A*_j_*), the greater risk of the operation (*f_hs_*) and when less protective measures are employed (*f_ps_*), the greater incorporation likelihood. Doctors and technicians performing ^131^I automatic dose fractionation got the d*_j_* of 47. In addition, they were responsible for ^99m^Tc elution, labelling and dose fractionation, and the corresponding d*_j_* of ^99m^Tc was 24 (the sum of 20, 2.0 and 2.0). The nurses injecting ^99m^Tc got the lowest d*_j_* of 4.0. Compared with nurses injecting ^99m^Tc only, doctors and technicians reached the higher “decision factor” (d*_j_*), and so probably received a greater committed effective dose due to a larger workload than nurses. Considering the hospital is a large and very busy provincial cancer center, the results are understandable. To balance the staffing and the workload to minimize the dose received by the workers are challenges to be addressed.

China is a country with a vast territory possessing numerous hospitals, and the level and scale of the hospitals, the workload of the workers and the protective measures employed in the field of nuclear medicine vary around the country. We can expect very substantial differences for d*_j_*, as well as the committed effective dose among hospitals and workers around the country. This study only investigated 18 nuclear medicine staff of one specialized hospital, and further investigation on the necessity of the internal monitoring for nuclear medicine staff in different areas and different levels of hospitals in China is urgently needed.

Additionally, considering that the decision factor assessment may overestimate the committed effective dose [21], internal monitoring work is needed to solve the problem of what dose from internal radiation the staff actually receive. Internal monitoring can be conducted in three ways: direct measurement of radionuclide in whole body or some organs, excreta or other biological samples analysis and air sampling analysis, respectively [6]. Compared with the external individual monitoring, the method of internal individual monitoring is diversified, and the result is easily influenced by many factors. The published literature reports [9,10,11,12,13,14,15,16,17,18,19,20] regarding internal exposure in the field of nuclear medicine mainly did research on commonly used nuclides ^131^I, ^99m^Tc and ^18^F, and among them, internal exposure induced by ^131^I were most studied and reported.

It is important to note that since the 1980s, the coverage of external individual monitoring of radiation workers in China has been increasing steadily [26], and China has also successively formulated and issued a series of standards [6,27,28] on internal individual monitoring, but for various reasons, internal individual monitoring has not been currently implemented in most areas of China. The 18 workers in the study wear under-apron thermoluminescent LiF (Mg, Cu, P) dosimeters on the chest when working in order to obtain the external dose, and the criterion used for external dose monitoring is GBZ 128-2002 [29]. Their recorded external doses were less than 1 mSv in a year. An investigation [9] showed that internal exposure caused by the inhalation of radioactive aerosol is an important part of occupational exposure of the nuclear medical staff, so we should attach great importance to it. Relevant research work on internal monitoring and dose assessment are needed [26]. Methods of internal monitoring that are suitable to Chinese situation are under in-depth exploration.

Some limitations in the study must be acknowledged. First, a work model of taking turns was employed in this nuclear medicine department to try to achieve a balanced workload among these workers. Daily operation records of each staff of all the year round are not well-kept, and we can only find records of the last two months. Hence it is difficult to get accurate activities of each operation and each worker. We have adopted the average values. Theoretically, the hospital should have accurate official records of radionuclides it purchased. Second, most of the patients with thyroid cancer were administered 3.7 × 10^9^ Bq^131^I, and only a few of them were administered a dose of 5.6 × 10^9^ Bq or 7.4 × 10^9^ Bq, we simplified the calculation and adopted a dose of 3.7 × 10^9^ Bq for all the patients when calculating, which caused certain underestimation. Third, after doctors or technicians performed ^99m^Tc dose fractionation, the ^99m^Tc labeled radiopharmaceutical were injected into the bodies of patients in minutes. Due to the radioactive decay of ^99m^Tc (T_1/2_ = 6.02 h), the activity of injection is slightly smaller than the activity of dose fractionation. Since minutes are much shorter than 6.02 h, we ignore the decay of ^99m^Tc in minutes and approximately consider that the activity of injection and dose fractionation are the same. In summary, it is rather difficult to obtain relatively accurate handling activity for each nuclide of each worker. Daily operation records of each staff member for all the year round are not well-preserved, and it is difficult to accurately estimate activities of each operation and each worker. Also, the criteria suggested by IAEA relate solely to inhalation of radionuclides, so other intake routes including ingestion of radionuclides are not taken into account in this study. Eating and drinking are strictly prohibited in China when handling radionuclides, but it may be necessary to confirm this.

## 5. Conclusions

The direct application of the criteria proposed in the IAEA Safety Guide RS-G-1.2 highlights the need for internal monitoring of all of the staff in the nuclear medicine department of the provincial cancer hospital. This is similar to the conclusions of previous published research, though the ratio may be higher, emphasizing the necessity of internal monitoring and importance of control of internal contamination. The study only investigated 18 nuclear medicine staff members of one Chinese hospital, and further investigation of the necessity for internal monitoring of nuclear medicine staff in different areas and different levels of hospitals in China is urgently needed. Due attention should be given to risk of internal contamination of the nuclear medicine workers and other related workers.

## Figures and Tables

**Table 1:** Dose coefficients for inhalation of 5 μm aerosols by workers (Sv·Bq^−1^) for the commonly handled radionuclides given by ICRP Publication No. 68 [24].

Radionuclides	e(g)*_j,inh_* (Sv·Bq^−1^)
^18^F	8.9 × 10^−11^
^67^Ga	2.8 × 10^−10^
^68^Ga	8.1 × 10^−11^
^89^Sr	1.4 × 10^−9^
^90^Y	1.6 × 10^−9^
^99m^Tc	2.0 × 10^−11^
^111^In	3.1 × 10^−10^
^123^I	1.1 × 10^−10^
^125^I	7.3 × 10^−9^
^131^I	1.1 × 10^−8^
^153^Sm	6.8 × 10^−10^
^2^°^1^Tl	7.6 × 10^−11^

**Table 2:** Standard handling safety factors based on the nature of the operation [8,25].

Operation	Handling Safety Factor (*f_hs_*)
Radioactive waste management	0.01
Elution	1
Labelling	1
Dose fractionation ^a^	1
Dose administration (injection)	1
Ventilation studies	1
Radioimmunoassay techniques	10

Notes: ^a^, Dose fractionation refers to the manual dose fractionation. The corresponding handling safety factor of ^131^I automatic dose fractionation was assigned the value of 0.1 on the basis of the actual situation in this study.

**Table 3:** Protection safety factors [8].

Protection Measure	Protection Safety Factor (*f_ps_*)
Glove box	0.01
Fume hood	0.1

**Table 4:** Collected information in the selected specialized hospital.

Staff	Work-Distribution	Work Model	Annual Activity (Bq)
Six nurses	^99m^Tc injection	Take turns, one person per week	1.2 × 10^13^
Three technicians and nine doctors	^99m^Tc elution	Take turns, one person per week	1.2 × 10^13^
	^99m^Tc labelling	Take turns, one person per week	1.2 × 10^13^
	^99m^Tc dose fractionation	Take turns, one person per week	1.2 × 10^13^
	^131^I automatic dose fractionation	Take turns, one person per batch patients	5.2 × 10^12^

**Table 5:** *f_hs_* and *f_ps_* values of operations performed in the selected specialized hospital.

Operation	Handling Safety Factor (*f_hs_*)	Protection Safety Factor (*f_ps_*)
^99m^Tc Elution	1	0.1
^99m^Tc Labelling	1	0.01
^99m^Tc Dose fractionation	1	0.01
^99m^Tc Dose administration (injection)	1	0.01
^131^I automatic dose fractionation	0.1	0.01

**Table 6:** d and D values of nuclear medicine staff in the selected specialized hospital.

Staff	Radionuclide	Operation	Annual Activity (Bq)	d*_j_*	D
nurse 1	^99m^Tc	Injection	2.0 × 10^12^	4.0	4.0
nurse 2	^99m^Tc	Injection	2.0 × 10^12^	4.0	4.0
nurse 3	^99m^Tc	Injection	2.0 × 10^12^	4.0	4.0
nurse 4	^99m^Tc	Injection	2.0 × 10^12^	4.0	4.0
nurse 5	^99m^Tc	Injection	2.0 × 10^12^	4.0	4.0
nurse 6	^99m^Tc	Injection	2.0 × 10^12^	4.0	4.0
doctor 1	^131^I	Dose fractionation	4.3 × 10^11^	47	71
	^99m^Tc	Elution	1.0 × 10^12^	20	
	^99m^Tc	Labelling	1.0 × 10^12^	2.0	
	^99m^Tc	Dose fractionation	1.0 × 10^12^	2.0	
doctor 2	The same as doctor 1				71
doctor 3	The same as doctor 1				71
doctor 4	The same as doctor 1				71
doctor 5	The same as doctor 1				71
doctor 6	The same as doctor 1				71
doctor 7	The same as doctor 1				71
doctor 8	The same as doctor 1				71
doctor 9	The same as doctor 1				71
technician 1	The same as doctor 1				71
technician 2	The same as doctor 1				71
technician 3	The same as doctor 1				71
